# Syndrome de la pince aorto-mésentérique sur cancer gastrique: à propos d’un cas

**DOI:** 10.11604/pamj.2022.42.217.27281

**Published:** 2022-07-20

**Authors:** Abdou Niasse, Papa Mamadou Faye, Abdourahmane Ndong, Serge Ramou Kuadjovi, Abdoulaye Leye, Ngoné Diaba Diack, Mohamadou Lamine Gueye, Ousmane Thiam, Mamadou Ndiaye, Ahmed Diouf, Ibrahima Sitor Souleymane Sarr, Yacine Seye, Alpha Oumar Toure, Mamadou Seck, Mamadou Cisse, Madieng Dieng

**Affiliations:** 1 Service de Chirurgie, Centre Hospitalier National de Pikine, Dakar, Sénégal,; 2 Service de Chirurgie Générale, Centre Hospitalier Universitaire Aristide Le Dantec, Dakar, Sénégal,; 3 Service de Chirurgie Générale, Hôpital Régional de Saint-Louis, Saint-Louis, Sénégal,; 4 Service de Médecine Interne, Centre Hospitalier National de Pikine, Dakar, Sénégal,; 5 Service de Chirurgie Générale, Centre Hospitalier Universitaire Dalal Jamm, Dakar, Sénégal

**Keywords:** Syndrome de la pince aorto-mésentérique, syndrome de Wilkie, gastro-jéjunostomie, cancer gastrique, cas clinique, Superior mesenteric artery syndrome, Wilkie´s syndrome, gastrojejunostomy, gastric cancer, case report

## Abstract

Responsable d´un tableau d´occlusion intestinale haute aiguë ou chronique, le syndrome de la pince aorto-mésentérique ou syndrome de Wilkie résulte de la compression du troisième duodénum entre l´artère mésentérique supérieure et l´aorte. La tomodensitométrie abdominale facilite le diagnostic. La dénutrition sévère est le principal facteur étiologique. Son traitement peut être médical par aspiration gastrique et nutrition parentérale. En cas d´échec, la chirurgie s´impose. Nous rapportons le cas d´un patient de 46 ans, tabagique, reçu pour vomissements bilieux et alimentaires post-prandiaux abondants. Il présentait un amaigrissement de 7% sur 6 mois. La fibroscopie digestive haute retrouvait une masse tumorale antro-pylorique non sténosante. L´histologie était en faveur d´un adénocarcinome tubuleux peu différencié de l´estomac. Le bilan d´extension était négatif et a permis de découvrir un syndrome de pince aorto-mésentérique avec un angle de 8°C. Il a bénéficié d´une nutrition parentérale pendant 10 jours suivie d´une gastrectomie polaire inférieure et d´une anastomose gastro-jéjunale sur anse en oméga. Les suites étaient simples. Une chimiothérapie adjuvante a été indiquée.

## Abstract (en)

Superior mesenteric artery syndrome or Wilkie syndrome is due to the compression of the third duodenum between the superior mesenteric artery and the aorta. It causes acute or chronic upper bowel occlusion. Abdominal CT scan facilitates the diagnosis. Severe malnutrition is its main etiological factor. Medical treatment can be based on aspiration of gastric contents and parenteral nutrition. If this fails, surgery is necessary. We here report the case of a 46-year-old patient, with a history of smoking, presenting with profuse postprandial bile and food vomiting. He had had weight loss of 7% over a period of 6 months. Upper GI endoscopy revealed non-stenotic antro-pyloric tumour mass. Histological examination showed poorly differentiated tubular gastric adenocarcinoma. Staging was without any peculiarity and allowed for the detection of superior mesenteric artery syndrome at an angle of 8°C. The patient received parenteral nutrition for 10 days, followed by inferior pole gastrectomy and gastrojejunal anastomosis (omega loop). The postoperative course was uneventful. Adjuvant chemotherapy was indicated.

## Introduction

Le syndrome de la pince mésentérique (SPAM) se définit par la compression de la troisième portion du duodénum (D3) entre l´artère mésentérique supérieure (AMS) en avant et le plan aorto- rachidien en arrière [1]. C´est une cause rare d´occlusion digestive haute. Il s´agit souvent de la conséquence d´un hypercatabolisme, d´une dénutrition sévère, mais aussi après une chirurgie de correction des déformations rachidiennes ou traction sur le mésentère [2,3]. Les signes cliniques les plus fréquents sont les douleurs abdominales, les vomissements bilieux et les nausées, souvent associés à une distension épigastrique [4,5]. Le traitement est avant tout médical. Il repose sur la mise en place d´une sonde naso-gastrique laissée en aspiration douce, la correction des troubles hydro-électrolytiques et la nutrition parentérale ou entéro-jéjunale [6]. La fonte de la graisse rétropéritonéale sur dénutrition sévère chez les cancéreux, est un facteur étiologique largement rapporté dans la littérature. Nous rapportons le cas d´un jeune adulte chez qui un syndrome de la pince aorto-mésentérique (SPAM) a été découvert lors du bilan d´extension d´un cancer gastrique. La particularité était cette association qui nécessita un traitement adéquat pour les 2 pathologies.

## Patient et observation

**Information du patient:** il s´agissait d´un patient de 46 ans, tabagique à raison de 10 paquets-année. Il a été amené en consultation en urgence pour une symptomatologie évoluant depuis 6 mois faite d´anorexie, de vomissements alimentaires et bilieux, d´épigastralgies et d´altération de l´état général avec amaigrissement chiffré à 7%.

**Résultats cliniques:** l´examen clinique retrouvait une tension artérielle à 10/09 mmHg, un BMI à 12 kg/m^2^, une déshydratation extracellulaire. L´abdomen était distendu avec clapotage en région épigastrique. Le reste de l´examen était sans particularités.

**Figure 1:**
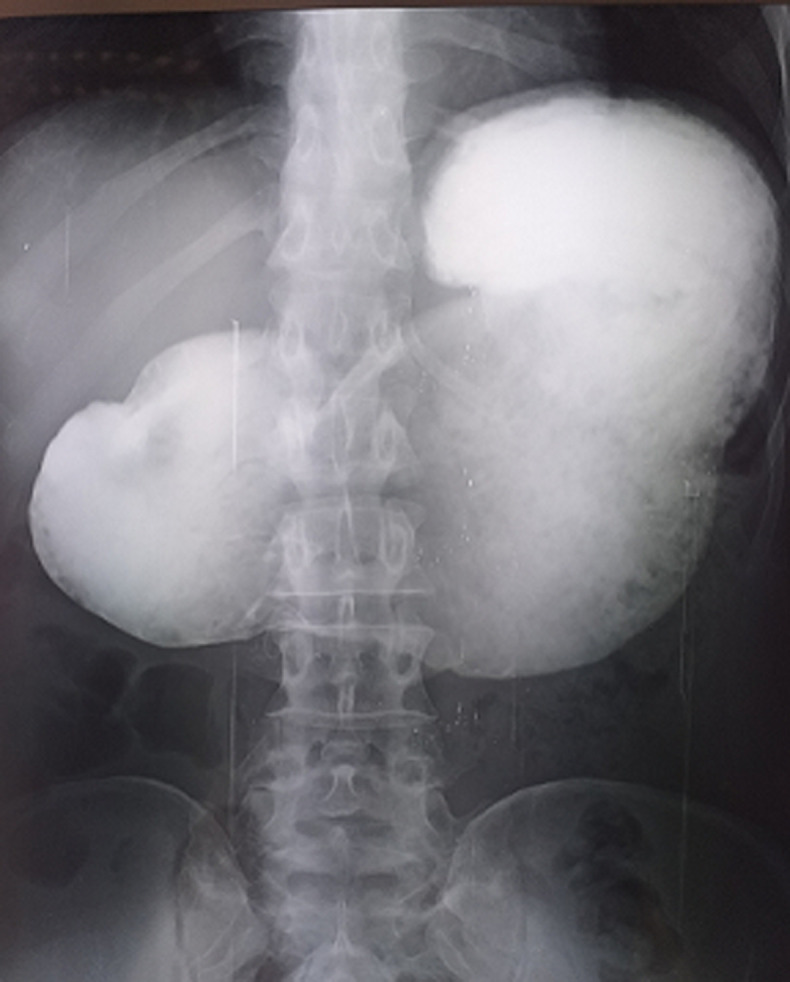
transit œsogastroduodénal montrant la dilatation gastrique en amont d’un arrêt du produit de contraste au niveau antral

**Figure 2:**
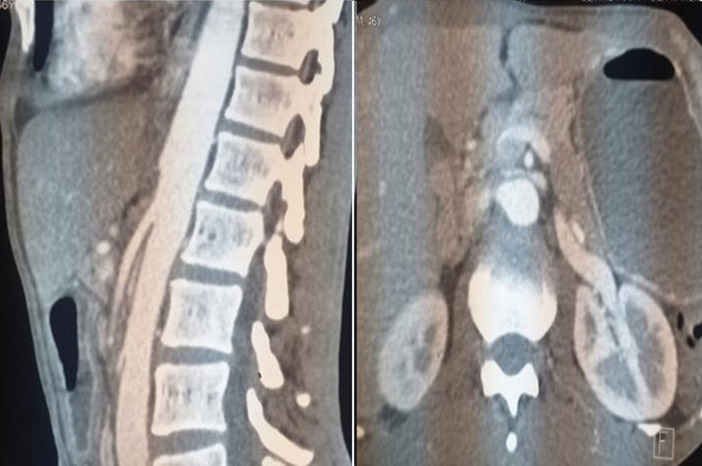
coupe scanographique sagittale et transversale montrant l’angle aorto-mésentérique mesuré à 8° et la distance aorte-artère mésentérique supérieure à 3,9 mm

**Démarche diagnostique:** la biologie montrait une urémie: 2,09 g/l, créatininémie à 41,5 mg/l, hyponatrémie à 122 mmol/l, hypokaliémie à 2,6 mmol/l et une anémie hypochrome microcytaire à 10,3 g/dl. Il n´y avait pas de cholestase clinico-biologique. Le transit œsogastroduodénal à la gastrographine a montré une dilatation gastrique en amont d´un arrêt linéaire du produit de contraste au niveau antro-pylorique (Figure 1). La fibroscopie digestive haute a montré une tumeur ulcérobourgeonnante antro-pylorique facilement franchissable dont l´histologie après biopsie retrouva un adénocarcinome tubuleux peu différencié infiltrant. La tomodensitométrie thoraco-abdomino-pelvienne a permis de retrouver, en dehors de la tumeur gastrique sans extension loco-régionale et à distance, une pince de la distance aorto-mésentérique mesurée à 3,9 mm et l´angle aorto-mésentérique mesuré à 8°C responsable d´une compression du 3^e^ duodénum avec stase liquidienne duodénale et gastrique en amont (Figure 2).

**Figure 3:**
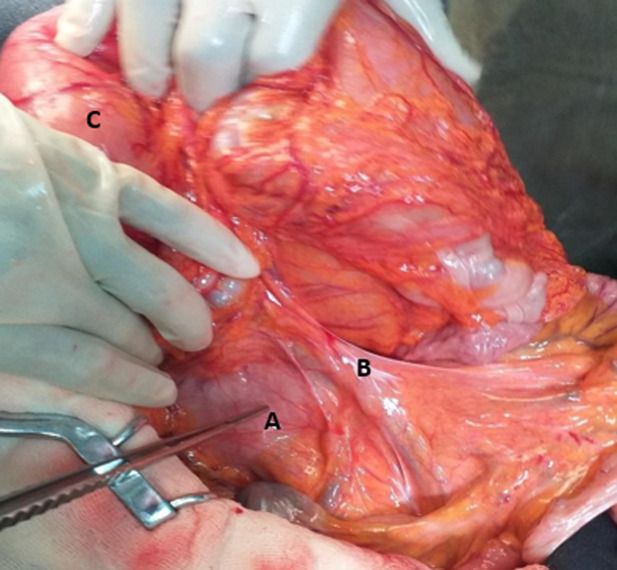
vue per-opératoire de la dilatation duodénale en amont de l’empreinte de l’artère mésentérique supérieure; A) 3^e^ duodénum dilaté; B) empreinte de l’artère mésentérique supérieure; C) estomac distendu

**Intervention thérapeutique et suivi:** un traitement médical a d´abord été entrepris avec mise en place d´une sonde naso-gastrique, apport hydroélectrolytique, nutrition parentérale hypercalorique et transfusion sanguine. Après dix jours de traitement, il y´a eu une amélioration clinique et une correction des troubles biologiques. La sonde gastrique ramenant en moyenne 1200 ml par jour. Après réunion de concertation pluridisciplinaire, il a été décidé et réaliser une laparotomie. Elle a permis de découvrir une masse tumorale antro-pylorique mobile de 3 cm, 2 adénopathies péri-duodénales et une importante distension gastrique et des 3 premières portions duodénales sans ascite ni carcinose péritonéale (Figure 3). Le foie était lisse. Il a été réalisé une gastrectomie polaire inférieure avec curage D1,5 puis rétablissement de la continuité digestive selon Finsterer sur anse en oméga sans dérivation duodénojéjunale. L´évolution fut favorable avec disparition des vomissements, diminution de la production quotidienne de la sonde gastrique (passer de 1200 ml à 250 ml), et reprise du transit au cinquième jour post-opératoire et l´alimentation orale a été autorisée à J5. Le patient fut sortant au septième jour post-opératoire. Une chimiothérapie adjuvante a été indiquée. Après 6 mois de suivi, il est resté asymptomatique.

**Consentement du patient:** le patient a donné son consentement éclairé pour la publication de ce cas.

## Discussion

Le SPAM a été décrit pour la première fois en 1842 par Carl Von Rokitansky. En 1927, Wilkie a publié la première série de 75 patients, depuis ce syndrome porte son nom [7]. Il est provoqué par une compression extrinsèque de la troisième portion du duodénum par l´artère mésentérique supérieure ou l´une de ses branches contre le plan aorto-rachidien. Cette obstruction peut être partielle ou complète, aiguë ou chronique, congénitale ou acquise [8]. Sa prévalence varie entre 0,013% et 0,78% et survient préférentiellement chez les jeunes patients de sexe féminin sans prédisposition raciale ou ethnique identifiée [9]. Sur le plan physiopathologique, ce syndrome est lié à un espace aorto-mésentérique et à un angle aorto- mésentérique réduit [10].

Chez notre patient, l´angle aorto-mésentérique était de 8° et l´espace aorto-mésentérique était de 3,9 mm. Cet angle est compris normalement entre 25° et 60° et la distance aorto-mésentérique est de 10 à 28 mm [1,4]. Plusieurs facteurs favorisent ces modifications anatomiques. À l´état normal, le duodénum est protégé par le tissu graisseux péri-vasculaire [10]. L´amaigrissement rapide entraine une diminution de l´épaisseur du tissu adipeux au niveau de l´espace aorto-mésentérique. Chez notre patient, la sévérité des vomissements et l´aspect non sténosant de la tumeur, laisse supposer que l´occlusion digestive était plutôt liée au SPAM. La dénutrition sévère liée à l´hypercatabolisme est probablement à l´origine de la fonte de la graisse péri-vasculaire. Ce qui a réduit l´angle aorto-mésentérique et entrainant la compression du duodénum et est à l´origine des vomissements. D´autres facteurs favorisants le SPAM ont été rapportés tels que la correction d´une scoliose, une hyperlordose rachidienne, une infirmité motrice cérébrale et des anomalies anatomiques telles qu´un ligament de Treitz hypertrophié ou anormalement court attirant la troisième portion duodénale vers le sommet de l´angle duodénojéjunal et favorisant ainsi la compression du segment digestif par l´artère mésentérique supérieure [8,10].

Néanmoins, 40,4% des cas surviennent sans facteur déclenchant évident [3,5,11]. Les symptômes sont variables et non spécifiques. L´installation peut être aigüe ou évoluer insidieusement en fonction de l´étiologie et de l´importance de l´obstruction duodénale. Les signes cliniques les plus fréquents sont les douleurs abdominales, les vomissements bilieux et les nausées, souvent associés à une distension épigastrique. Ils sont aggravés par les repas et le décubitus dorsal, soulagés par le décubitus latéral gauche et la position assise [8,10,12]. Cette symptomatologie, retrouvée chez notre patient, pose le problème de diagnostic différentiel et évoque plutôt une tumeur gastrique, un ulcère gastro-duodénal sténosant ou une atrésie duodénale chez l´enfant [12,13]. L´association cancer antro-pylorique et SPAM chez notre patient, font douter sur l´origine de la symptomatologie. Néanmoins, l´imagerie et l´endoscopie ont révolutionné le diagnostic. La radiographie standard et le transit œsogastroduodénal confirment l´obstruction digestive haute. La fibroscopie montre la stase gastro-duodénale et la Tomodensitométrie (TDM) calcule l´angle entre l´artère mésentérique supérieure et l´aorte qui est réduit de 7° à 22°, et distance aorto-mésentérique qui est réduite aussi et mesure entre 2 - 8 mm [4,7]. Le traitement de la SPAM est d´abord médical, et consiste en la mise en place d´une sonde nasogastrique pour provoquer une décompression de l´estomac et du duodénum, mettre le patient en position latérale gauche, et surtout compenser les désordres hydroélectrolytiques et instaurer une alimentation hypercalorique double, entérale par une sonde naso-jéjunale et parentérale [1,5,7].

Le succès dans ce cas avoisine les 72% mais avec des récidives de l´ordre de 30% [14]. En cas d´échec, la chirurgie s´impose. Chez notre patient, la tumeur entretenait la dénutrition avec l´hypercatabolisme. Ce qui aggrave le SPAM et ceci constitue alors un cercle vicieux. Ce qui justifia l´indication chirurgicale. Le traitement chirurgical consiste en la réalisation soit d´une dérivation par gastro- jéjunostomie ou duodéno-jéjunostomie [1,3], réalisable par voie laparoscopique [1], ou modifier les conditions anatomiques en faisant une mobilisation et décroisement de l´angle duodéno-jéjunal en positionnant le jéjunum à droite de l´AMS après section du muscle de Treitz selon le procédé de Strong, les meilleurs résultats obtenus sont ceux de la duodéno-jéjuno-anastomose [7,15]. L´absence d´ictère chez notre patient et la présence de la tumeur gastrique ont constitué les éléments clés de l´indication de la gastrectomie polaire inférieure que nous avions jugé assez suffisante pour contourner l´obstacle duodénal sans entraver l´écoulement de la bile, mais également traiter le cancer.

## Conclusion

Etant rare et pouvant survenir à tout âge, le syndrome de la pince aorto-mésentérique est à évoquer devant toute occlusion haute chez un patient en dénutrition sévère notamment cancéreux. La tomodensitométrie et le transit digestif facilitent son diagnostic. Le traitement médical visant à corriger la dénutrition et la déshydratation, peut s´avérer inefficace et fera recourir à la chirurgie.

## References

[1] Bonnet JP, Louis D, Foray P (1995). La pince aorto-mésentérique supérieure primitive. Arch Pédiatr.

[2] Roy A, Gisel JJ, Roy V, Bouras EP (2005). Superior Mesenteric Artery (Wilkie´s) Syndrome as a Result of Cardiac Cachexia. Journal of General Internal Medicine.

[3] Zadegan F, Lenoir T, Drain O, Dauzac C, Leroux R, Morel E (2007). Syndrome de la pince aorto-mésentérique après correction d´une déformation rachidienne: À propos d´un cas et revue de la littérature. Rev Chir Ortho et Rép Appareil Moteur.

[4] Unal B, Aktas A, Kemal G, Bilgili Y, Güliter S, Daphan C (2005). Superior mesenteric artery syndrome: CT and ultrasonography findings. Diagn Interv Radiol Ank Turk.

[5] Andaloussi S, Mahmoudi A, Khattala K, Bouabdallah Y (2019). Le syndrome de la pince aorto-mésentérique: une cause rare d´obstruction duodénale. Pan African Medical Journal.

[6] Kadji M, Naouri A, Bernard P (2006). Syndrome de la pince aorto-mésentérique: à propos d´un cas. Ann de chir.

[7] Welsch T, Büchler MW, Kienle P (2007). Recalling Superior Mesenteric Artery Syndrome. Digestive Surgery.

[8] Mathenge N, Osiro S, Rodriguez II, Salib C, Tubbs RS, Loukas M (2014). Superior mesenteric artery syndrome and its associated gastrointestinal implications. Clin Anat.

[9] Kalouche I, Léturgie C, Tronc F, Bokobza B, Michot F, Pons P (1991). The superior mesenteric artery syndrome: apropos of a case and review of the literature. Ann Chir.

[10] Kwan E, Lau H, Lee F (2004). Wilkie´s syndrome. Surgery.

[11] Tidjane A, Tabeti B, Benmaarouf N, Boudjenan N, Bouziane C, Kessai N (2014). Le syndrome de la pince aorto-mésentérique: rare, mais pensez-y. Pan African Medical Journal.

[12] Zaraket V, Deeb L (2015). Wilkie´s Syndrome or Superior Mesenteric Artery Syndrome: Fact or Fantasy. Case Rep Gastroenterol.

[13] Fall M, Bâ PA, Baba FT, Alassane PM, Ngom G (2014). Le syndrome de la pince aorto-mésentérique chez l´enfant: à propos d´un cas primitive. Pan African Medical Journal.

[14] Shin MS, Kim JY (2013). Optimal Duration of Medical Treatment in Superior Mesenteric Artery Syndrome in Children. J Korean Med Sci.

[15] Merrill KF Superior Mesenteric Artery Syndrome Treatment & Management.

